# Construct Failure in an Atypical Femoral Fracture treated with Intramedullary Nailing: A Case Report

**DOI:** 10.5704/MOJ.1403.008

**Published:** 2014-03

**Authors:** L Bonifacio, P Syson

**Affiliations:** Philippine Orthopedic Center, Maria Clara St. corner Banawe Ave., Quezon City, Philippines; Philippine Orthopedic Center, Maria Clara St. corner Banawe Ave., Quezon City, Philippines

## Abstract

The aim of this paper is to document a rare case of construct failure in a 68-year old Filipina who sustained an atypical femoral fracture (AFF) in her left subtrochanteric area. The patient previously had a 40-month history of alendronate 70mg + vitamin D 5600u therapy for osteoporosis and underwent closed intramedullary nailing for the AFF. Six months postoperatively, she began to experience progressive pain in her operated thigh. Radiographs revealed a broken nail at the proximal screw hole and non-union of the AFF. The patient was treated with exposure of the fracture site, removal of the broken device, exchange intramedullary nailing, and iliac bone grafting. She had radiographic and clinical union and was full weight bearing after three months.

## Introduction

A typical femoral fractures (AFFs) have been reported after prolonged intake of bisphosphonates (BPs). There has been a steady increase in published evidence associating BPs and AFFs, but a causal relationship has not yet been established. The updated case definition of an AFF is a fracture located along the femoral diaphysis from just distal to the lesser trochanter to just proximal to the supracondylar flare, with at least four of five major features present. These features are: (1) minimal or no trauma, (2) fracture line originates at the lateral cortex and is substantially transverse, (3) complete fractures may be associated with a medial spike while incomplete fractures involve only the lateral cortex, (4) noncomminuted or minimally comminuted, and (5) localized periosteal or endosteal thickening of the lateral cortex (“beaking” or “flaring”)^1^.

Delayed fracture healing is a recognized minor feature in AFFs, which may or may not be present in a given case. If delayed healing or non-union does occur, it may be complicated further by the presence of a broken implant. Literature still lacks a standardized treatment regimen. The aim of this paper is to document a rare case of construct failure in a 68-year old Filipina who sustained an AFF in her left subtrochanteric area.

## CASE REPORT

A 68 year old Filipina was admitted by the senior author on December 12, 2010 due to sudden occurrence of severe pain on her previously operated left thigh while she was trying to stand up from a chair. The pain was so severe that she immediately called for help and asked to be brought to the emergency room for consultation. Apparently, the patient had been ignoring a slight on and off pain on her left thigh for one week previously. Upon further questioning, it emerged that the patient had undergone closed intramedullary nailing around six months ago (June 2, 2010) at another institution for an atypical femoral fracture AFF. The initial injury was sustained when she suddenly felt severe left thigh pain that caused her to fall and land on her buttocks, while walking casually at home.

Prior to this, the patient had a 40-month history of alendronate 70mg + vitamin D 5600u once-a-week therapy for osteoporosis that was diagnosed by a central dual energy X-ray absorptiometry (DEXA). She was managed by her primary orthopedic surgeon with immediate cessation of BPs and closed intramedullary nailing. The patient was noted to have minimal callus formation on radiographs at three months postoperatively and was advised weight-bearing as tolerated while using a walker. However, the patient was lost to follow-up and was already bearing full weight with no symptoms until six months postoperatively, when the present condition started.

Radiographs at the emergency room revealed a broken intramedullary nail and atrophic non-union of the AFF. The senior author operated on the patient, using a lateral decubitus position on a radiolucent table through the interval between the tensor fascia lata and vastus lateralis. Upon opening of the fracture site, fibrous tissue was found at the fracture ends. The broken device was removed, an 11 mm diameter Simplified Universal Nail. It had a transverse break at the proximal static interlocking screw hole, while all four screws were intact. After using a rongeur to remove the fibrous tissue and obtain bleeding edges at the fracture site,the canal was over reamed to 13 mm. and a 12 mm diameter reconstruction nail with two distal transverse and two proximal neck directed interlocking screws were inserted. The excised bone chips were then onlayed onto the fracture site and supplemented with bone graft harvested from the ipsilateral iliac crest. Routine wound closure by layers was done, without application of any drain.

No pharmacologic supplementation was prescribed. At three months post-operatively, radiographic and clinical union was confirmed. Full weight-bearing without assistive device was allowed, and the patient went on to heal uneventfully.

## Discussion

Initial treatment of an AFF requires immediate discontinuation of BPs, plus fracture stabilization. Since primary bone healing is compromised, secondary healing through callus formation is preferred, which is best accomplished by a full-length intramedullary device. The contralateral femur must also be assessed for impending AFF, and the patient should be started on medical therapy, which always includes calcium and vitamin D supplementation^1^.

Delayed fracture healing is a recognized minor feature in AFFs, which may or may not be present in a given case. This delay may be explained by suppressed bone remodelling secondary to the anti-resorptive action of BPs. Studies have shown that the initial formation of fracture callus and woven bone are not affected by BPs. However, to complete the fracture repair process, a remodelling phase with cortical bone healing must occur. This is suppressed by BPs due to inhibition of the coupled action of osteoblasts and osteoclasts^2^.

Weil et al described the outcome of surgically treated femur fractures associated with prolonged BP use in 15 patients with 17 AFFs. They found that only 54% healed without necessitating secondary procedures such as nail dynamization, exchange nailing to a larger diameter nail, or even complete revision to a different fixation device (i.e. blade plate). This is quite low compared with the very successful (98-99%) healing rate reported in studies dealing with regular femoral fractures that were fixed with intramedullary nailing. They attributed this higher failure rate to a possible impaired healing process in AFFs because they did not note any change in surgical technique compared when they treated regular femoral fractures. According to them, in their series of 1,500 regular femoral fractures, they were able to maintain a less than 2% nonunion rate^3^.

AFFs should be followed-up closely, and some reports have even recommended pharmacologic supplementation postoperatively. Recently, Giannotti et al. reported a case of pseudarthrosis in a 65-year old woman with AFF. She underwent intramedullary nailing in 2010, but was subsequently re-admitted in 2012 due to persistent pain. Radiographs revealed an intact implant but with an atrophic non-union. The patient was then treated with trephination of the outbreak of non-union, insertion of a new intramedullary nail, and supplementation with strontium ranelate. The fracture united uneventfully after three months ^4^.

In our case, we did not give pharmacologic supplementation because evidence is still lacking regarding what specific drug would be beneficial for each patient. Some reports suggest combined anabolic/catabolic agents such as strontium ranelate, while others suggest recombinant parathyroid hormone such teriparatide, but an actual comparison with each other or with no pharmacologic supplementation is not yet available. Fortunately for our patient, we were still able to attain union by just freshening of the fracture site, removal of the broken device, exchange intramedullary nailing, and iliac bone grafting.

Interestingly, atypical fractures are now being reported in other areas besides the subtrochanteric femur. Moon et al described forearm fractures in two women receiving longterm bisphosphonate therapy, with no history of significant trauma. The first woman presented with a transverse fracture of the proximal ulnar shaft and incomplete fractures of bilateral distal femurs. She was treated with open reduction and plating of the ulna and closed nailing of both femurs. The second woman presented with an incomplete fracture of the proximal to middle radial shaft. She was treated with a long-arm cast. Both women did not receive pharmacologic supplementation, yet both also had an uneventful course of healing ^5^.

**Fig. 1: Antero-posterior radiograph of left proximal femur showing non-union of the AFF with the broken intramedullary nail.**
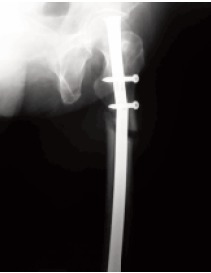


**Fig. 2: The broken device, an 11 mm diameter Simplified Universal Nail, with a transverse break at the proximal static interlocking screw hole, while all 4 screws were intact.**
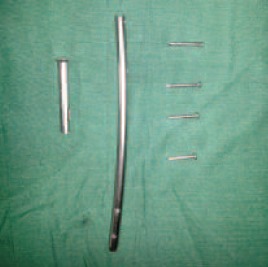


**Fig. 3: Antero-posterior radiograph of left proximal femur showing the healed AFF.**
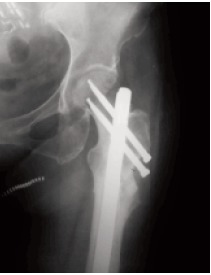


## Conclusion

AFFs are now more commonly recognized due to increasing awareness. Atypical fractures have also begun to be reported in other areas besides the subtrochanteric femur. Initial treatment of an AFF requires immediate discontinuation of BPs, plus fracture stabilization. The case presented in this report though, shows that correct diagnosis and proper treatment according to current recommendations do not guarantee a good outcome. If delayed healing or non-union do occur, it may be complicated further by the presence of a broken implant. An effective and consistent protocol has not yet been defined for AFFs with delayed healing or nonunion. Pharmacologic supplementation may or may not be given, with no clear evidence yet to support what kind of drug to give and for which subgroup of patients. Nevertheless, emerging reports of cases managed successfully may help guide treatment of these difficult injuries.
